# Rheumatic Fever and Its Treatment

**Published:** 1876-02

**Authors:** 


Rheumatic Fever and its Treatment.—At a recent meeting of the British Medical Association at Edinburgh, Dr. James Johnston brought forward for consideration with regard to the causation of rheumatic fever, the non conversion of starchy food into glucose in consequence of irritation from improper food or from exposure to cold and damp, and the conversion in the cæcum of the undigested starch into lactid acid, which being absorbed produced the phenomena of the disease. In regard to the treatment to be observed he advocated the use of bi-carbonate of soda, given in enema, instead of potash. In twenty cases so treated the temperature had gone down to below the normal state in the course of seven days. In a preceding paper Dr. Russell Reynolds had shown how early relief had been obtained, not only from pain, but from the abnormal heat by the use of the tincture of the perchloride of iron, forty-four per cent. of first attacks being convalescent within the first week, whilst of those in the second, third, or fourth attacks, forty-two per cent. recovered within the same period. In the discussion that followed the reading of Dr. Johnston's paper, Dr. Long Fox, of Bristol, stated that he was more content with the practice of frequent blistering of the joints than he was with anything else, especially when this was combined with the use of alkalies. Mr. Meacham, who could speak from experience, believed that carbonate of soda was one of the very best remedies we have in medicine for rheumatic fever or rheumatism. He would not have any objection to take two drachms of corbonate of soda at any time when he suspected an attack was approaching.—*Brit. Med. Jour.*

